# Aminoalcohol-Induced Activation of Organophosphorus Hydrolase (OPH) towards Diisopropylfluorophosphate (DFP)

**DOI:** 10.1371/journal.pone.0169937

**Published:** 2017-01-13

**Authors:** Dandan Li, Yunze Zhang, Haitao Song, Liangqiu Lu, Deli Liu, Yongze Yuan

**Affiliations:** 1 Hubei Key Laboratory of Genetic Regulation and Integrative Biology, School of Life Sciences, Central China Normal University, Wuhan, Hubei, P. R. China; 2 Department of Biochemistry, School of Basic Medical Sciences, Hubei University of Medicine, Shiyan, Hubei, P.R. China; 3 Key Laboratory of Pesticide & Chemical Biology, Ministry of Education, College of Chemistry, Central China Normal University, Wuhan, Hubei, P. R. China; Kermanshah University of Medical Sciences, ISLAMIC REPUBLIC OF IRAN

## Abstract

Aminoalcohols have been addressed as activating buffers for alkaline phosphatase. However, there is no record on the buffer activation regarding organophosphorus hydrolase (OPH). Here we reported the activating effects of aminoalcohols on OPH-catalyzed hydrolysis of diisopropylfluorophosphate (DFP), an analog molecule of G-type warfare agents. The kinetic parametors *k*_cat_, *V*_max_ and *k*_cat_/*K*_m_ in the OPH reaction were remarkably increased in the buffers (pH 8.0, 25°C) containing aminoalcohols with C2 between nitrogen (N) and oxygen (O) in their structures, including triethanolamine (TEA), diethanolamine, monoethanolamine, 1-amino-2-propanol, 2-amino-2-methyl-1-propanol, and triisopropanolamine. In contrast, much lower or no rate-enhancing effects were observed in the adding of amines, alcohols, amine/alcohol mixtures, or 3-amino-1-propanol (C3 between N and O). The 300 mM TEA further increased DFP-degrading activities of OPH mutants F132Y and L140Y, the previously reported OPH mutants with desirable activities towards DFP. However, the treatment of ethylenediaminetetraacetate (EDTA) markedly abolished the TEA-induced activation of OPH. The product fluoride effectively inhibited OPH-catalyzed hydrolysis of DFP by a linear mixed inhibition (inhibition constant *K*_i_ ~ 3.21 mM), which was partially released by TEA adding at initial or later reaction stage. The obtained results indicate the activation of OPH by aminoalcohol buffers could be attributed to the reduction of fluoride inhibition, which would be beneficial to the hydrolase-based detoxification of organophosphofluoridate.

## Introduction

**Fig 1:**
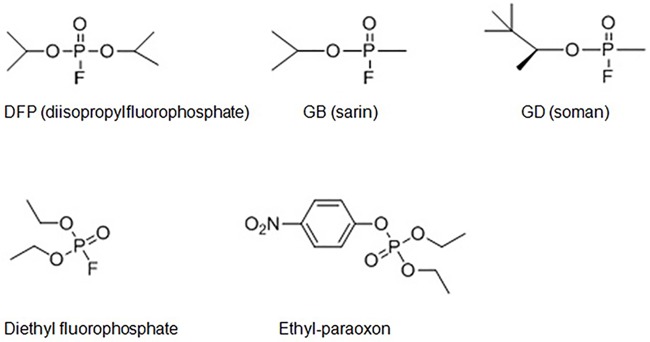
Molecular structures of DFP, GB, GD, diethyl fluorophosphate, and paraoxon (ethyl-paraoxon).

Organophosphorus hydrolase (OPH; EC 3.1.8.1), encoded by the identical *opd* genes from soil bacterium *Pseudomonas diminuta* MG and *Flavobacterium* sp. ATCC 27551, has been characterized as a typical phosphotriesterase (PTE) with a rather broad substrate specificity [1–4]. This enzyme, in the form of native or recombinant protein, has been documented to catalyze the hydrolysis of a wide variety of acetylcholinesterase (AchE) inhibitors, the chemical compounds highly toxic to mammalian organisms [5,6]. The substrates of OPH include organophosphate insecticides paraoxon, parathion, methylparathion, coumaphos, and diazinon, as well as potent nerve agents sarin, soman, and their analog diisopropylfluorophosphate (DFP) (Fig 1).

OPH is one of the best characterized OP hydrolases and the catalysis mechanism has been intensively proposed based on the protein structures. The crystal structure of OPH revealed the decisive contribution of binuclear metal center in the active site to the hydrolytic activity towards different OPs [4,7–12]. The binuclear metal center, comprised of either two equivalents of Zn^2+^ in the native enzyme or mixed metal ions Cd^2+^, Co^2+^, Mn^2+^, and Ni^2+^ in the metal-substituted catalysts, has been illustrated to generate the activated water molecules that initiate nucleophilic attack on the phosphorus atom of the substrate, resulting in the cleavage of phosphoester bond and the release of leaving group [2–4,8–10]. In addition, the rate of OPH-catalyzed hydrolysis depends on the substrate. The OPH exhibited much lower rates to DFP hydrolysis, as compared to its best substrate paraoxon [2,13,14]. Up to now, all the efforts to enhance the enzyme activity against DFP have focused on the generation of OPH mutants through directed evolution and the further protein engineering [2,3,8,15–17]. The contribution of buffer environment to enhancing catalytic abilities of OPH (DFP hydrolase) has been rarely studied.

The buffer and solvent species have been proposed to remarkably influence enzyme activities. The alkaline phosphatase (ALP), a typical group of phosphatases (EC 3.1.3.1), exhibited much better performance on phosphomonoesters hydrolysis in the tris and imidazole buffers [18,19]. The various aminoalcohol derivatives, including 2-(ethylamino)ethanol, *N*-methylethanolamine, diethanolamine, and triethanolamine, have been verified as activators to enhance bovine intestine ALP (BIALP) activity to degrade *p*-nitrophenyl phosphate (pNPP) [20]. ALPs have been classified to the metalloenzyme, and the reported activators are nitrogen-containing organic compounds that probably act as phophoacceptor and/or suitable ligands for the critical metal ions. Recently, two sets of synthetic nitrogen-containing organic materials, Zn^2+^-azamacrocyclic complexes and oxime substituted β-cyclodextrin derivatives, were illustrated as the novel non-enzyme catalysts accelerating the hydrolysis of paraoxon and cyclosarin (GF) [21,22]. These reports suggested the activating effects of hydroxyl amine nitrogen derivatives on the particular OP hydrolysis. In the present study, we proposed the activation of DFP hydrolysis by aminoalcohols and kinetically addressed this hypothesis.

**Fig 2:**
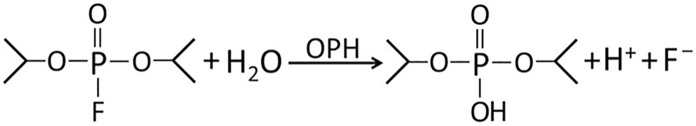
OPH-catalyzed hydrolysis of DFP.

Previous reports have described many chemical compounds capable of inhibiting OP hydrolase activities. Dementon-S has been identified as a noncompetitive inhibitor against paraoxon hydrolysis [16,23]. Paraoxon at the concentration beyond 400 μM was reported to induce a specific substrate inhibition in the OpdA-catalyzed hydrolysis of this OP compound [14]. The fluoride, typically in the form of anion F^−^, is an effective inhibitor of metalloenzymes, including peroxidases [24], laccase [25], ureases [26], arginase [27], purple acid phosphatases (PAPs) [28,29], and OP hydrolases [14]. The inhibition of fluoride on paraoxon hydrolysis has been kinetically assessed for OpdA and OPH, both enzymes requiring dinuclear metal centre to activate hydroxide in the initiation of catalysis [14]. Fluoride is one of products released during OPH-catalyzed hydrolysis of DFP (Fig 2), thus the effect of aminoalcohol on fluoride inhibition is also an interesting issue to address.

**Fig 3:**
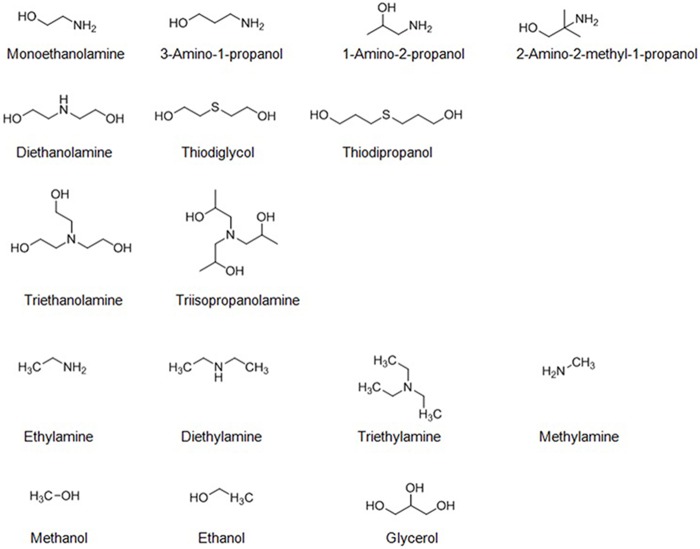
Molecular structures of aminoalcohols, aminoalcohol analogs, amines and alcohols examined in this study.

In the present study, we compared the effects of various aminoalcohols, amines, and alcohols (Fig 3) on OPH activity against DFP by kinetics studies. To discuss the mechanism on OPH activation by aminoalcohols, the kinetics of fluoride inhibition on the OPH-catalyzed DFP hydrolysis was also analyzed in the absence and in the presence of triethanolamine.

## Materials and Methods

### Bacterial strains, plasmids, and materials

*Flavobacterium* sp. ATCC 27551, recently identified as *Sphingobium fuliginis* [30], was obtained from the American Type Culture Collection (Manassas, VA, USA) for the *opd* gene cloning. The *E*. *coli* strains DH5α (TaKaRa, Otsu, Japan) and BL21(DE3) (Novagen, Darmstadt, Germany) were used for recombinant plasmid amplification and protein expression, respectively. The cloning vector pUC118 was purchased from TaKaRa (Otsu, Japan), and the vector pET-28 (Novagen), which introduces a His6-tag (His-tag^™^; Novagen) at the N-terminus, was used for gene expression. Isopropyl thio-β-dgalactoside (IPTG), ampicillin, kanamycin, imidazole, and Tris Base were purchased from Ameresco (Tully, NY, USA). The restriction enzymes *Eco*RI and *Hin*dIII and the other reagents used in gene cloning were products of TaKaRa (Otsu, Japan). All chemicals were of analytical grade and purchased from Sinopharm Chemical Reagent Corp. (Shanghai, China). Diisopropylfluorophosphate (DFP), the substrate for OPH catalysis, was synthesized in our laboratory and identified by Nuclear Magnetic Resonance (NMR) spectroscopy (S1 Fig).

### Cloning and site-directed mutagenesis of OPH

*Sphingobium* sp. ATCC 27551 was cultured in SP medium, as described by Ohmori et al. [31], and the genomic DNA was extracted using a bacterial DNA extraction kit (Tiangen Biotech, Beijing, China) according to the manufacturer's instructions. The full-length *opd* gene was amplified from the genomic DNA by polymerase chain reaction (PCR) to construct plasmid pUC-opd as described by Ohmori et al [31]. Using the plasmid pUC-opd as templates, the gene encoding OPH without N-terminus 29 amino acids was PCR-amplified with the designated primers (S1 Table), and overlap-extension PCR was performed to generate the mutants F132Y, L140Y, and F132Y/L140Y with primers listed in S1 Table. The PCR products were purified, digested with *Eco*RI and *Hin*dIII, and then ligated with vector pET-28. The constructed plasmids were transformed into *E*.*coli* DH5α for positive clone screening and DNA sequencing.

### Expression and purification of recombinant OPH

The pET-opd(m) plasmids encoding His6-tagged OPH (wild type and mutants) were transformed into *E*.*coli* BL21(DE3) cells to express recombinant enzymes with IPTG induction. After 18 h induction at 18°C, the cells were collected by centrifugation, disrupted by sonication, and the target enzyme, validated by sodium dodecyl sulfate polyacrylamide gel electrophoresis (SDS-PAGE), was purified by a Ni-NTA His-bind^™^ Resin column (Novagen) according to the manufacturer’s instructions. The protein concentration was determined using the method of Bradford [32], and the purified proteins were stored at -20°C until further use.

### Kinetic analysis of OPH-catalyzed hydrolysis of DFP

The rate of OPH-catalyzed hydrolysis of DFP was measured by monitoring the release of fluoride (F^−^) with an F^−^-specific electrode (Thermo Orion, USA) as described previously [2, 3]. The reaction mixture (10 ml) containing 50 mM Tris-HCl (pH 8.0), 100 μM ZnCl_2_, 1–50 mM DFP, and 10 nM enzyme sample was used to determine DFP hydrolysis rate at 25°C in the absence and presence of aminoalcohols, amines and alcohols. 1.0 M HCl was used to adjust pH 8.0 for all the assays. The initial velocity (*V*) (μM/min), defined as F^−^ production rate in the first 5 min with a deduction of non-enzymatic hydrolysis rate of DFP, was plotted against DFP concentrations (0, 2, 4, 6, 8, 10, 20, 30, 40, and 50 mM) and made fitting to the Michaelis-Menten equation (hyperbola profile) using the Enzyme Kinetics Module from GraphPad Prism (version 5.0). The kinetic parameters, maximum velocity (*V*_max_) and Michaelis constant (*K*_m_), were calculated by the plots of 1/*V* against 1/[DFP] with changing DFP concentrations according to Lineweaver-Burk equation, i.e. 1/*V* = 1/*V*_max_ + (*K*_m_/*V*_max_)**·**(1/[DFP]). The *k*_cat_ values were calculated as *V*_max_/[E] ([E] = 10 nM) and also made fitting to the Michaelis-Menten equation using GraphPad Prism (version 5.0). The specificity constant (*k*_cat_/*K*_m_) was obtained from the determined *k*_cat_ and *K*_m_. To investigate if the alcohol and amine groups must be present in the same molecule regarding the additive-induced rate-enhancing effects, an one plus one mixture of an alcohol and a simple amine (ethylamine, diethylamine, triethylamine, or methylamine) at 300 mM (final concentration) was added to OPH reaction solution mentioned above, and the kinetic parameters *k*_cat_, *V*_max_ and *K*_m_ were determined. The changes of kinetic parameters (*k*_cat_, *V*_max_ and *K*_m_) for OPH-catalyzed hydrolysis of DFP after adding analog molecules for MEA, DEA and TEA were also investigated. The MEA analogs investigated were 3-amino-1-propanol, 1-amino-2-propanol and 2-amino-2-methyl-1-propanol, the DEA analogs were thiodiglycol and thiodipropanol, and the TEA analog was triisopropanolamine. The concentration of each additive used in this study was 300 mM, and the other conditions were the same as described above. To investigate the role of metal center (Zn^2+^) in the aminoalcohol effects, *k*_cat_ values were determined for the wild-type and mutant OPH (F132Y, L140Y and F132Y/ L140Y) incubated with 50 mM ethylenediaminetetraacetate (EDTA) for 2 h at 25°C, and the kinetic analysis was conducted with the described reaction buffer (pH8.0) in the absence and presence of 300 mM triethanolamine.

### Kinetic analysis of fluoride inhibition

The kinetic analysis of sodium fluoride (NaF) inhibition was conducted in the absence and presence of triethanolamine (100, 200, and 300 mM). At each triethanolamine concentration, the substrate DFP was incubated in 10 ml of assay buffer (50 mM Tris-HCl (pH 8.0) and 100 μM ZnCl_2_) with 10 nM OPH and different concentrations of inhibitor NaF (0, 1, 2, and 3 mM). The enzyme was pre-incubated at 4°C with NaF for 2 h before the initiation of assays at 25°C. The apparent *V*_max_ and *K*_m_ in the inhibitory effects with or without triethanolamine were calculated from 1/*V* vs 1/[S] plots with changing DFP concentrations (2, 4, 6, 8, 10, 20, 30, 40, and 50 mM), as described in Lineweaver-Burk equation. In the analysis of fluoride inhibition, the *k*_cat_ was calculated by the apparent *V*_max_ and total enzyme concentration (10 nM), and the specific constant (*k*_cat_/*K*_m_) was calculated by *k*_cat_ and *K*_m_. The inhibition constant (*K*_i_) for NaF in the OPH catalysis was calculated by the replot of apparent *K*_m_ obtained from the double-reciprocal plots against inhibitor NaF concentrations. To investigate buffering capacity of aminoalcohol for fluoride inhibitor in OPH-catalyzed hydrolysis of DFP, 500 μL of NaF stocking (300 mM) was added to 50 mL of TEA-free or -containing reaction solution (50 mM Tris-HCl/pH 8.0, 100 μM ZnCl_2_, initial OPH concentration = 10 nM, and initial DFP concentration = 20 mM) at the end of the first reaction stage (0–5 minutes), i.e. at the beginning of the second reaction stage (5–10 minutes; defined as "later reaction stage"), and the dependence of DFP hydrolysis velocity on reaction time (0–10 minutes) was recorded once per minute at 25°C. Similarly, at the beginning of the mentioned later reaction stage (5–10 minutes), a defined volume of TEA-HCl stocking (3 M; pH 8.0) was added to 50 mL of enzymatic solution (50 mM Tris-HCl/pH 8.0, 100 μM ZnCl_2_, initial OPH concentration = 10 nM, and initial DFP concentration = 20 mM) to the final TEA concentration 100, 200, and 300 mM, and these reaction systems were used to further evaluate the aminoalcohol-induced OPH activation under an obvious fluoride inhibition condition.

## Results

### Effects of aminoalcohols on the OPH-catalyzed hydrolysis of DFP

**Fig 4:**
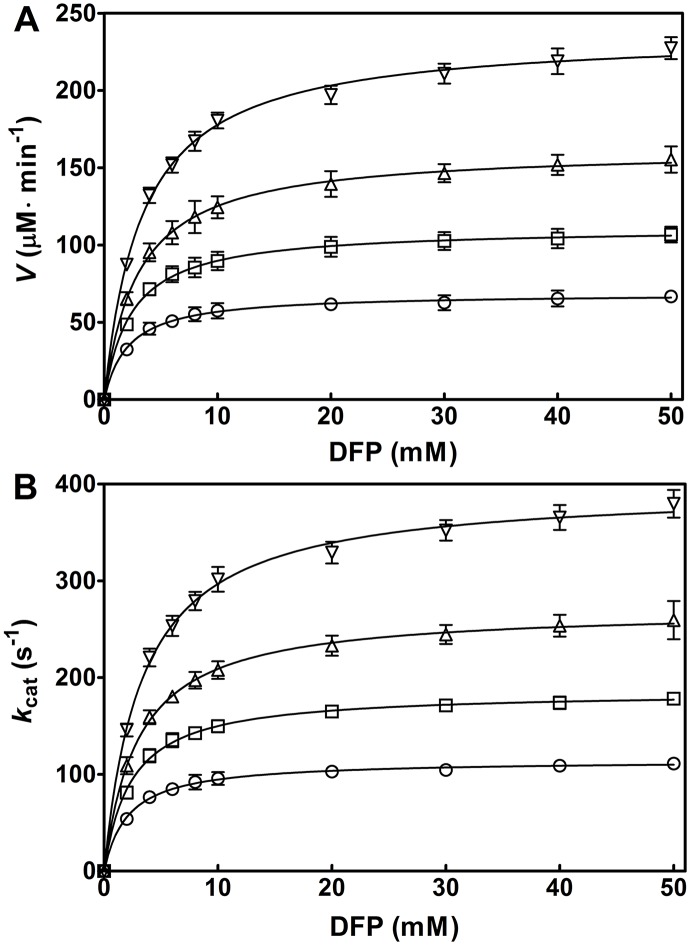
Kinetic analysis of OPH-catalyzed hydrolysis of DFP in the presence of 300 mM aminoalcohols. The reaction was carried out in the presence of 100 μM ZnCl_2_, at pH 8.0 and 25°C. The initial enzyme concentration, [E], is 10 nM for every reaction. The initial velocity (*V*) (A) and the *k*_cat_ (*V*/[E]) (B) are respectively plotted against DFP concentrations (0, 2, 4, 6, 8, 10, 20, 30, 40, and 50 mM). Solid line represents the best fit of the Michaelis-Menten equation using the Enzyme Kinetics Module from GraphPad Prism (version 5.0). Symbols for the buffers: control (in the absence of aminoalcohols), ○; monoethanolamine (MEA), □; diethanolamine (DEA), △; and triethanolamine (TEA), ▽. Data are expressed as the mean±SD of three independent experiments.

To investigate the effects of aminoalcohols on the OPH-catalyzed hydrolysis of DFP, we first made kinetic analysis of OPH in the hydrolysis of DFP with various aminoalcohols. As shown in Fig 4, the OPH-catalyzed hydrolysis of DFP displayed Michaelis-Menten-type saturation behavior in the presence of the investigated aminoalcohols. Compared to the control, the initial velocity (*V*) for the enzymatic hydrolysis of DFP was markedly increased with 300 mM triethanolamine (TEA), diethanolamine (DEA), and monoethanolamine (MEA), at pH 8.0 and 25°C (Fig 4A). The order of aminoalcohols for the activation efficiency was TEA > DEA > MEA. In the presence of the three aminoalcohols, the similar results were obtained for the change of *k*_cat_ (*V*/[E]) in the OPH reactions (Fig 4B). In contrast, little activating effects of the aminoalcohols were observed in non-enzymatic hydrolysis of DFP (data not shown). The results indicate that all the examined aminoalcohols activate the OPH-catalyzed hydrolysis of DFP.

**Fig 5:**
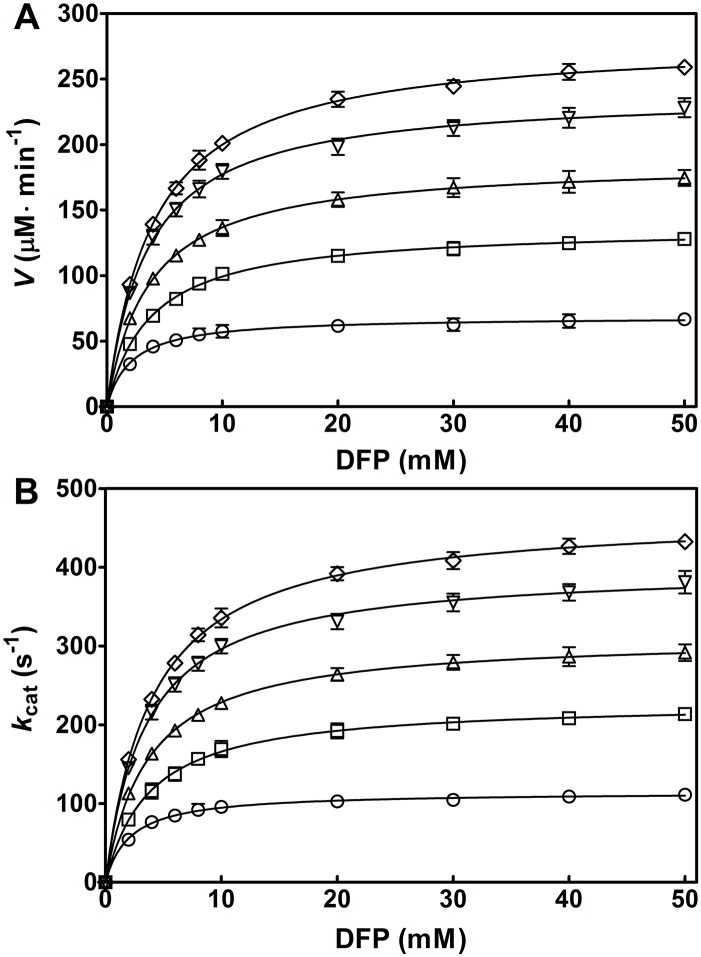
Kinetic analysis of OPH-catalyzed hydrolysis of DFP with increasing triethanolamine (TEA) concentrations. The reaction was carried out in the presence of 100 μM ZnCl_2_, at pH 8.0 and 25°C. The initial enzyme concentration, [E], is 10 nM for every reaction. The initial velocity (*V*) (A) and the *k*_cat_ (*V*/[E]) (B) are respectively plotted against DFP concentrations (0, 2, 4, 6, 8, 10, 20, 30, 40, and 50 mM). Solid line represents the best fit of the Michaelis-Menten equation using the Enzyme Kinetics Module from GraphPad Prism (version 5.0). Symbols for TEA concentration (mM): 0, ○; 100, □; 200, △; 300, ▽; and 400 ◇. Data are expressed as the mean±SD of three independent experiments.

Further, TEA, the best activator in the present study, was chosen to assess the effect of aminoalcohol concentration on the OPH activation. The increase in the apparent *V* (Fig 5A) and *k*_cat_ (*V*/[E]) (Fig 5B) was clearly observed with varying TEA concentrations (100–400 mM). The activation folds were gradually increased with the given TEA concentrations. The activation of OPH by TEA was also kinetically fit to the Michaelis-Menten equation (Fig 5). All the plots in Fig 5 showed saturated profiles, thus the associated kinetic parameters of OPH, including *k*_cat_, *V*_max_, *K*_m_ and *k*_cat_/*K*_m_, were determined separately (Table 1). In the given concentration range (100–400 mM), the *k*_cat_ values were elevated with increasing aminoalcohol concentrations: the value at 400 mM TEA was 477 ± 6 s^−1^, which was 206% of that at 100 mM (231 ± 7 s^−1^); the value at 400 mM DEA was 312 ± 13 s^−1^, which was 204% of that at 100 mM (153 ± 8 s^−1^); and the value at 400 mM MEA was 209 ± 10 s^−1^, which was 146% of that at 100 mM (143 ± 10 s^−1^). The similar results were obtained in the changes of *V*_max_ and *k*_cat_/*K*_m_ with the increasing aminoalcohol concentrations (Table 1).

**Table 1:** Kinetic parameters of OPH in the DFP hydrolysis in the presence of aminoalcohols.

Conc. (mM)	*k*_cat_×10^−2^ (s^-1^)	*V*_max_ (μM min^-1^)	*K*_m_ (mM)	*k*_cat_/*K*_m_ (mM^-1^ s^-1^)
Control	1.17 ± 0.04	70.1 ± 0.1	3.01 ± 0.49	41.17 ± 6.79
Triethanolamine				
100	2.31 ± 0.07	138.5 ± 4.3	4.00 ± 0.51	59.87 ± 8.26
200	3.14 ± 0.10	188.3 ± 6.3	3.81 ± 0.53	85.59 ± 11.19
300	4.19 ± 0.14	251.1 ± 8.5	4.06 ± 0.39	105.58 ± 12.16
400	4.77 ± 0.06	285.8 ± 3.9	4.32 ± 0.49	112.98 ± 11.35
Diethanolamine				
100	1.53 ± 0.08	91.8 ± 4.6	3.05 ± 0.04	50.40 ± 3.05
200	2.37 ± 0.08	141.6 ± 4.5	3.42 ± 0.32	70.60 ± 7.92
300	2.77 ± 0.20	165.8 ± 12.0	3.16 ± 0.04	87.41 ± 5.30
400	3.12 ± 0.13	186.9 ± 7.9	3.35 ± 0.03	93.22 ± 4.71
Monoethanolamine				
100	1.43 ± 0.10	85.5 ± 5.7	3.18 ± 0.36	45.31 ± 4.02
200	1.59 ± 0.08	95.3 ± 4.9	3.19 ± 0.33	50.21 ± 3.90
300	1.92 ± 0.04	115.2 ± 2.5	2.80 ± 0.14	69.26 ± 5.00
400	2.09 ± 0.10	125.6 ± 5.8	3.15 ± 0.29	66.46 ± 2.57

The reaction was carried out in specific aminoalcohol buffers containing 100 μM ZnCl_2_, at pH 8.0 and 25°C. The initial enzyme concentration was 10 nM and the DFP concentrations were defined as 0, 2, 4, 6, 8, 10, 20, 30, 40, and 50 mM. Data are expressed as the mean±SD of three independent experiments.

### Effects of alcohols and amines on the OPH-catalyzed hydrolysis of DFP

To evaluate the contribution of hydroxyl and amino groups in the aminoalcohols to the activation of OPH, we made kinetic analysis of OPH in the hydrolysis of DFP with various alcohols (hydroxyl donor) and amines (amino donor). The 300 mM alcohols, including ethanol, glycerol, and methanol, had little effects on the kinetic parameters of OPH in the DFP hydrolysis (Table 2), indicating that these alcohols could not activate OPH-catalyzed hydrolysis of DFP. Table 3 described the kinetic parameters of OPH in the DFP hydrolysis with varying concentrations (100–300 mM) of ethylamine, diethylamine, triethylamine, and methylamine. Compared to the control (117 ± 4 s^-1^), the *k*_cat_ values for the OPH-catalyzed hydrolysis of DFP were slightly increased by the four amines at 300 mM, reaching 180 ± 8, 163 ± 12, 148 ± 9, and 193 ± 10 s^-1^, respectively (Table 3). The orders of these amines for the efficiency in the OPH activation were methylamine, ethylamine, diethylamine, and triethylamine. The similar results were obtained in the change of *V*_max_. However, the activation of OPH by these amines was not remarkable compared to that by the corresponding aminoalcohols. On the other hand, the *K*_m_ values also slightly increased with increasing concentrations of amines. The increasing rate of *K*_m_ was very similar to that of *k*_cat_. Thus, the ratio of *k*_cat_ to *K*_m_ (*k*_cat_/*K*_m_) exhibited no obvious increase at any amine concentrations, indicating the poor ability of the amines to activate OPH. Additionally, a mixture of ethanol and a simple amine (ethylamine, diethylamine, triethylamine, or methylamine) at 300 mM had no more higher ability to activate OPH, as compared to the amine alone (Table 4). The similar results were also obtained in such one plus one mixtures of the amines and alcohols (methanol and glycerol) (data not shown). Hence, the activating effects of additives observed in this study might require the coexistence of alcohol and amine groups in the same molecule.

**Table 2:** Kinetic parameters of OPH in the DFP hydrolysis in the presence of 300 mM alcohols.

Alcohol	*k*_cat_×10^−2^ (s^-1^)	*V*_max_ (μM min^-1^)	*K*_m_ (mM)	*k*_cat_/*K*_m_ (mM^-1^s^-1^)
Control	1.17 ± 0.04	70.1 ± 0.1	3.01 ± 0.49	41.17 ± 6.79
Ethanol	1.23 ± 0.01	73.9 ± 0.7	3.08 ± 0.16	40.25 ± 2.39
Glycerol	1.15 ± 0.02	68.7 ± 1.0	3.38 ± 0.14	34.12 ± 1.74
Methanol	1.23 ± 0.01	73.3 ± 0.6	3.18 ± 0.19	38.75 ± 2.40

Except the alcohol additives, the reaction conditions and statistics analysis were the same as described in Table 1.

**Table 3:** Kinetic parameters of OPH in the DFP hydrolysis in the presence of amines.

Conc. (mM)	*k*_cat_×10^−2^ (s^-1^)	*V*_max_ (μM min^-1^)	*K*_m_ (mM)	*k*_cat_/*K*_m_ (mM^-1^ s^-1^)
Control	1.17 ± 0.04	70.1 ± 0.1	3.01 ± 0.49	41.17 ± 6.79
Ethylamine				
100	1.26 ± 0.11	75.6 ± 0.6	4.01 ± 0.33	32.01 ± 2.92
200	1.52 ± 0.12	91.1 ± 1.2	4.21 ± 0.12	36.24 ± 1.50
300	1.80 ± 0.08	107.4 ± 0.4	4.56 ± 0.31	39.73 ± 2.93
Diethylamine				
100	1.21 ± 0.07	72.2 ± 0.7	3.97 ± 0.53	31.60 ± 4.48
200	1.45 ± 0.11	86.6 ± 0.9	4.53 ± 0.41	32.56 ± 3.43
300	1.63 ± 0.12	97.7 ± 0.5	4.67 ± 0.40	35.48 ± 3.40
Triethylamine				
100	1.14 ± 0.08	68.4 ± 0.5	3.90 ± 0.46	30.28 ± 4.20
200	1.26 ± 0.07	75.5 ± 1.9	4.04 ± 0.60	32.56 ± 4.64
300	1.48 ± 0.09	88.6 ± 2.4	4.52 ± 0.62	33.93 ± 4.36
Methylamine				
100	1.49 ± 0.11	89.0 ± 0.5	3.95 ± 0.24	37.91 ± 2.47
200	1.79 ± 0.13	107.3 ± 0.6	4.26 ± 0.16	42.19 ± 1.83
300	1.93 ± 0.10	115.3 ± 1.2	4.44 ± 0.11	43.43 ± 1.44

Except the amine additives, the reaction conditions and statistics analysis were the same as described in Table 1.

**Table 4:** Changes in kinetic parameters of OPH towards DFP hydrolysis after adding a mixture of alcohol and amine.

Mixture of alcohol and amine	*k*_cat_×10^−2^ (s^-1^)	*V*_max_ (μM min^-1^)	*K*_m_ (mM)
Control	1.17 ± 0.04	70.1 ± 0.1	3.01 ± 0.49
Ethanol + ethylamine	1.95 ± 0.12	117.0 ± 1.4	4.32 ± 0.25
Ethanol + diethylamine	1.78 ± 0.21	105.5 ± 2.6	4.41 ± 0.32
Ethanol + triethylamine	1.55 ± 0.14	93.1 ± 2.8	4.45 ± 0.22
Ethanol + methylamine	2.16 ± 0.27	128.8 ± 3.1	4.17 ± 0.20

The alcohol (ethanol) and amine concentrations used in this study were 300 mM, and the other reaction conditions and statistics analysis were the same as described in Table 1.

### Changes of kinetic parameters for OPH-catalyzed hydrolysis of DFP after adding analog molecules for MEA, DEA and TEA

The changes of kinetic parameters for OPH-catalyzed hydrolysis of DFP after adding analog molecules for MEA, DEA and TEA were further investigated, including thiodiglycol, thiodipropanol, and aminoalcohols with more than two carbon atoms between nitrogen (N) and oxygen (O). As shown in Table 5, even at 300 mM, 3-Amino-1-propanol, a C3 aminoalcohol with three carbon atoms between N and O, exhibited little ability to enhance OPH catalysis rate. In contrast, at the same concentration, the other aminoalcohols with more than two carbon atoms in structure but only two carbon atoms between N and O, including 1-amino-2-propanol (C3), 2-amino-2-methyl-1-propanol (C4), and triisopropanolamine (C9), all exhibited rate-enhancing effects on the OPH-catalyzed hydrolysis of DFP (Table 5). Triisopropanolamine (tertiary amine), an analog molecule of TEA, had approximately 85% activating effect of TEA, and the two MEA analog molecules, 1-amino-2-propanol and 2-amino-2-methyl-1-propanol (primary amines), had similar activating effects of MEA. Comparatively, the only one extra carbon atom between N and O sharply reduced the activating effects of aminoalcohols, suggesting the critical role of atom distance between amino-N and hydroxyl-O in such aminoalcohol-induced activation. Based on this, a similar system without N but with S (thiodiglycol and thiodipropanol) was also exploited in the present study. As shown in Table 5, at 300 mM, thiodiglycol with two carbon atoms between sulfur (S) and O, rather than thiodipropanol with just one more carbon atom, remarkably activated OPH-catalyzed hydrolysis of DFP, reaching at a comparable level of DEA.

**Table 5:** Changes of kinetic parameters for OPH-catalyzed hydrolysis of DFP after adding analog molecules of MEA, DEA and TEA.

Additives	*k*_cat_×10^−2^ (s^-1^)	*V*_max_ (μM min^-1^)	*K*_m_ (mM)
Control	1.17 ± 0.04	70.1 ± 0.1	3.01 ± 0.49
3-Amino-1-propanol	1.42 ± 0.11	84.9 ± 4.7	4.61 ± 0.22
1-Amino-2-propanol	1.95 ± 0.10	117.1 ± 3.8	2.92 ± 0.19
2-Amino-2-methyl-1-propanol	2.11 ± 0.12	126.5 ± 3.3	2.95 ± 0.20
Triisopropanolamine	3.61 ± 0.23	215.8 ± 6.4	4.21 ± 0.25
Thiodiglycol	2.95 ± 0.34	177.3 ± 5.9	3.97 ± 0.31
Thiodipropanol	1.51 ± 0.27	91.2 ± 4.5	4.77 ± 0.21

The MEA analogs with more than two carbon atoms in structure are 3-amino-1-propanol, 1-amino-2-propanol, and 2-amino-2-methyl-1-propanol. The DEA analogs with N atom replaced by S are thiodiglycol and thiodipropanol. The TEA analog with more than two carbon atoms in structure is triisopropanolamine. The concentration of each additive used in this study was 300 mM, and the other reaction conditions and statistics analysis were the same as described in Table 1.

### Effects of TEA on the catalytic activities of OPH mutants towards DFP in the absence and presence of EDTA

**Fig 6:**
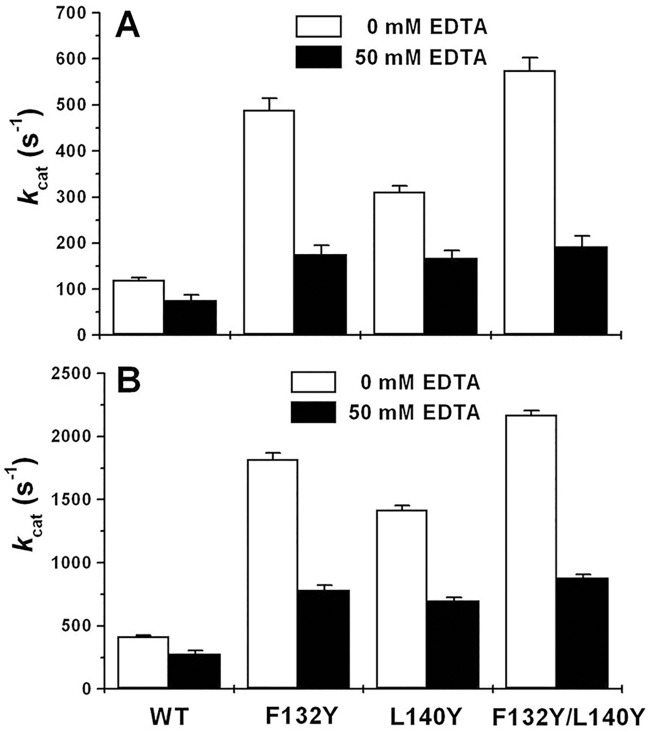
Kinetic analysis for EDTA inhibition of OPH activities towards DFP in the addition of 0 mM (A) and 300 mM (B) triethanolamine (TEA). Except for the TEA and EDTA concentrations particularly mentioned, the reaction conditions and statistics analysis were the same as described in Fig 4.

As reported by Watkins et al. [2] and Gopal et al. [3], OPH mutants F132Y and L140Y exhibited most desirable DFP-degrading activities, approximately 3–5 folds higher than the wild-type enzyme. However, these results were obtained in HEPES and ammonium carbonate buffers without any aminoalcohols. In the present study, to confirm the activating effects of aminoalcohol, we kinetically evaluated the OPH mutants towards DFP hydrolysis in the presence of 300 mM TEA. As shown in Table 6, 300 mM TEA markedly activated the OPH mutants concerning DFP hydrolysis. The apparent *k*_cat_ and *k*_cat_/*K*_m_ of double mutant F132Y/L140Y were increased to 2170 ± 40 s^-1^ and 541.7 ± 12.6 mM^-1^s^-1^ at 300 mM TEA, respectively 2.7- and 1.9-folds higher than those at 0 mM TEA (Table 6). The similar results were also obtained for the single-point mutants F132Y and L140Y. However, the treatments of 50 mM EDTA predominantly decreased *k*_cat_ values of OPH mutants concerning DFP hydrolysis (Fig 6A) and to a large extent abolished the activating effects of TEA on the DFP-degrading activities of OPH mutants (Fig 6B).

**Table 6:** Kinetic parameters of OPH mutants in the DFP hydrolysis with or without triethanolamine (TEA).

OPH	TEA (mM)	*k*_cat_×10^−2^ (s^-1^)	*V*_max_ (μM min^-1^)	*K*_m_ (mM)	*k*_cat_/*K*_m_ (mM^-1^ s^-1^)
WT	0	1.2 ± 0.0	70.1 ± 0.2	3.0 ± 0.5	41.2 ± 6.8
F132Y	0	4.9 ± 0.2	351.8 ± 17.6	3.2 ± 0.3	153.4 ± 4.9
L140Y	0	3.1 ± 0.1	237.4 ± 11.9	3.2 ± 0.3	97.3 ± 5.1
F132Y/L140Y	0	5.8 ± 0.3	412.1 ± 20.6	3.1 ± 0.1	185.8 ± 11.5
WT	300	4.2 ± 0.1	251.1 ± 8.5	4.1 ± 0.4	105.6 ± 12.2
F132Y	300	18.2 ± 0.5	1257.2 ± 63.8	4.2 ± 0.3	432.5 ± 10.9
L140Y	300	14.2 ± 0.4	847.5 ± 42.4	4.5 ± 0.3	319.6 ± 10.3
F132Y/L140Y	300	21.7 ± 0.4	1483.4 ± 75.8	4.0 ± 0.2	541.7 ± 12.6

The reaction conditions and statistics analysis were the same as described in Table 1.

### Fluoride inhibition of wild-type OPH in the absence and presence of TEA

**Fig 7:**
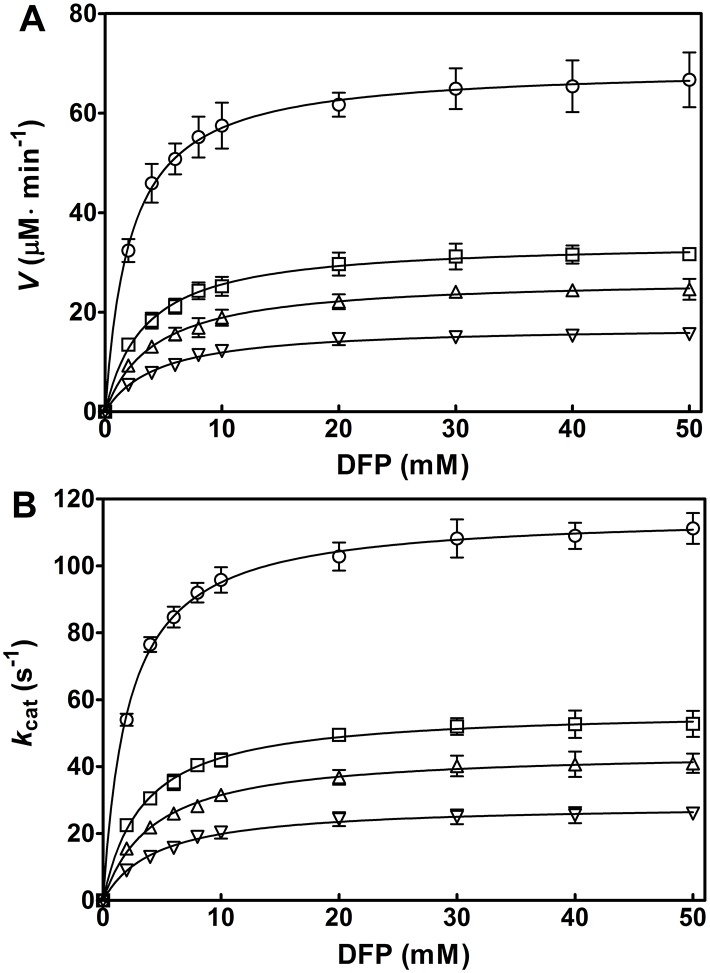
Kinetic analysis of fluoride inhibition of OPH-catalyzed DFP hydrolysis in the absence of triethanolamine. The reaction was carried out in the presence of 100 μM ZnCl_2_, at pH 8.0 and 25°C. The initial enzyme concentration, [E], is 10 nM for every reaction. The initial velocity (*V*) (A) and the *k*_cat_ (*V*/[E]) (B) at 0, 1, 2, and 3 mM NaF are respectively plotted against DFP concentrations (0, 2, 4, 6, 8, 10, 20, 30, 40, and 50 mM). Solid line represents the best fit of the Michaelis-Menten equation using the Enzyme Kinetics Module from GraphPad Prism (version 5.0). Symbols for NaF concentration (mM): 0, ○; 1, □; 2, △; and 3, ▽. Data are expressed as the mean±SD of three independent experiments.

**Fig 8:**
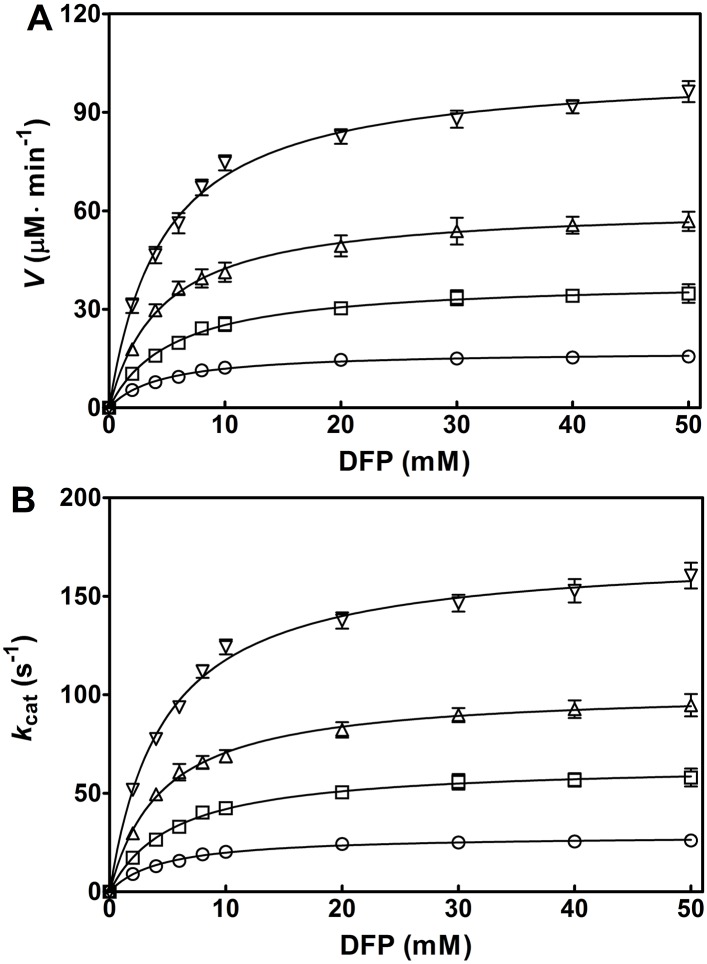
Kinetic analysis of fluoride inhibition of OPH-catalyzed DFP hydrolysis in the presence of triethanolamine. The reaction was carried out in 0 mM (○), 100 mM (□), 200 mM (△), and 300 mM (▽) TEA-HCl buffer (pH 8.0 and 25°C) containing 100 μM ZnCl_2_ and 3 mM NaF. The initial enzyme concentration, [E], is 10 nM for every reaction. The initial velocity (*V*) (A) and the *k*_cat_ (*V*/[E]) (B) are respectively plotted against DFP concentrations (0, 2, 4, 6, 8, 10, 20, 30, 40, and 50 mM). Solid line represents the best fit of the Michaelis-Menten equation using the Enzyme Kinetics Module from GraphPad Prism (version 5.0). Data are expressed as the mean±SD of three independent experiments.

Inhibition studies on the hydrolysis of DFP were performed using sodium fluoride (NaF) as the inhibitor against the wild-type OPH with Zn^2+^ as the active-site metal. The product (fluoride) inhibition of OPH in the absence of TEA was kinetically fit to the Michaelis-Menten equation (Fig 7). All the plots in Fig 7 showed saturated profiles, thus the associated apparent kinetic parameters of OPH, including apparent *K*_m_, *V*_max_ and *k*_cat_/*K*_m_, were determined separately (Table 7). As shown in Table 7, the apparent *V*_max_ and *k*_cat_/*K*_m_ of OPH for DFP hydrolysis gradually decreased with increasing NaF concentrations. This inhibitory effect of NaF was observed in the investigated buffers with 0, 100, 200, and 300 mM TEA (Table 7), suggesting a specific product inhibition of OPH by fluoride. The apparent *K*_m_ values were obviously elevated with increasing NaF concentrations at any TEA conditions, indicating a relatively poor affinity of DFP for OPH in the presence of fluoride inhibitor. At 3 mM NaF, the kinetics of OPH-catalyzed hydrolysis of DFP under TEA conditions were fit to the Michaelis-Menten equation (Fig 8), and the saturated profiles of all the plots allowed for the separate determination of apparent kinetic parameters for OPH (Table 7). In the absence of TEA, the apparent *V*_max_ and *k*_cat_/*K*_m_ were decreased to 17.2 ± 0.2 μM min^-1^ and 6.45 ± 0.94 mM^-1^s^-1^, respectively, at 3 mM NaF. In contrast, the adding of TEA increased the *V*_max_ and *k*_cat_/*K*_m_ values at the NaF treatment. As shown in Table 7, in the presence of 300 mM TEA, the apparent *V*_max_ and *k*_cat_/*K*_m_ at 3 mM NaF were 106.1 ± 1.1 μM min^-1^ and 36.23 ± 5.07 mM^-1^s^-1^, respectively, approximately 4.5-folds higher than that in the absence of TEA. At any concentration of inhibitor fluoride, the markedly increase in the kinetic parameters (*V*_max_ and *k*_cat_/*K*_m_) for the OPH-catalyzed hydrolysis of DFP were also observed in the TEA treatments (Table 7).

**Table 7:** Kinetic parameters of wild-type OPH in the DFP hydrolysis in fluoride inhibition with or without triethanolamine.

TEA (mM)	NaF (mM)	*K*_m_ (mM)	*V*_max_ (μM min^-1^)	*k*_cat_/*K*_m_ (mM^-1^s^-1^)
0	0	2.95 ± 0.04	70.2 ± 0.6	51.19 ± 6.12
	1	2.99 ± 0.09	33.1 ± 0.7	18.49 ± 5.48
	2	3.57 ± 0.02	25.5 ± 0.6	11.93 ± 3.95
	3	4.45 ± 0.01	17.2 ± 0.2	6.45 ± 0.94
100	0	3.52 ± 0.03	147.4 ± 0.6	69.90 ± 8.77
	1	4.23 ± 0.07	70.4 ± 0.2	27.79 ± 5.56
	2	4.87 ± 0.07	54.3 ± 0.6	18.62 ± 4.64
	3	5.58 ± 0.06	39.2 ± 0.2	11.73 ± 1.76
200	0	3.26 ± 0.08	201.6 ± 0.3	103.27 ± 11.65
	1	3.87 ± 0.12	116.6 ± 1.1	50.32 ± 4.01
	2	4.54 ± 0.08	88.5 ± 1.3	32.55 ± 2.53
	3	5.04 ± 0.09	63.9 ± 1.6	21.17 ± 1.23
300	0	3.49 ± 0.05	263.5 ± 1.4	126.09 ± 7.42
	1	4.06 ± 0.08	181.8 ± 1.0	74.78 ± 4.98
	2	4.58 ± 0.06	131.2 ± 0.7	47.84 ± 2.87
	3	4.89 ± 0.10	106.1 ± 1.1	36.23 ± 5.07

At each TEA concentration, the inhibitor sodium fluoride (NaF) (0, 1, 2, and 3 mM) were used for kinetic analysis. The reaction conditions and statistics analysis were the same as described in Table 1.

**Fig 9:**
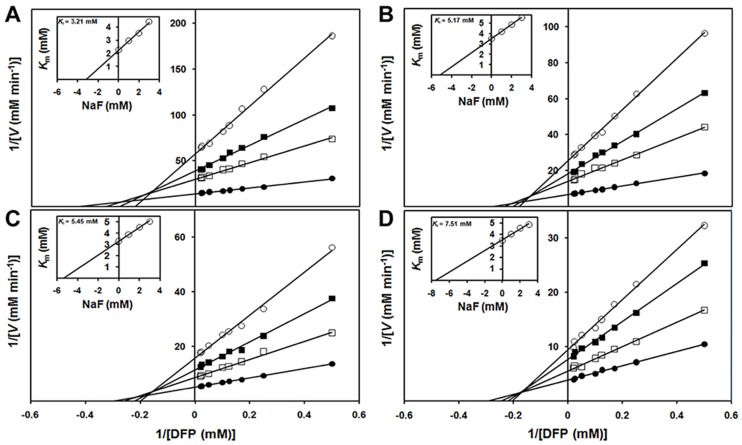
Analysis of inhibition kinetic constant (*K*_i_) for fluoride (NaF) in OPH-catalyzed hydrolysis of DFP. The inhibition analysis of fluoride was performed at the increasing triethanolamine (TEA) concentrations: 0 mM (A), 100 mM (B), 200 mM (C) and 300 mM (D). The TEA-HCl buffer (pH 8.0) at 25°C contained 100 μM ZnCl_2_, the initial enzyme of 10 nM and 0–3 mM NaF. At each TEA concentration, the 1/*V* was plotted against 1/[DFP] under 0 mM (●), 1 mM (□), 2 mM (■), and 3 mM (○) NaF conditions. Inset shows the replot of the apparent *K*_m_ obtained from the double-reciprocal plot versus inhibitor (NaF) concentrations. Each point in the plot is the average of triplicate determination with the experimental error less than 10%.

Fig 9 illustrated the Lineweaver-Burk plots of Zn^2+^-liganded OPH in the presence of 0, 100, 200, and 300 mM TEA. At each TEA concentration, three different inhibitor (NaF) concentrations were used, ranging from 1.0 to 3.0 mM. The double-reciprocal plot for the inhibitor-associated OPH catalysis at specific TEA concentration showed convergent lines crossing approximately at the same point in the second quadrant (Fig 9), indicating a typical linear mixed inhibition. The inset illustrated the replot of the apparent *K*_m_ obtained from double-reciprocal plot against inhibitor (NaF) concentration, indicating the inhibition constant *K*_i_ for NaF by the horizontal intercept. The results obtained showed a linear curve (Fig 9, inset), indicating that the fluoride, in the form of NaF, is a linear inhibitor of OPH activity towards DFP in the concentration range tested. The calculated *K*_i_ values for NaF in the presence of 0, 100, 200, and 300 mM TEA were 3.21 (Fig 9A), 5.17 (Fig 9B), 5.45 (Fig 9C), and 7.51 mM (Fig 9D), respectively. The *K*_i_ for NaF in the OPH-catalyzed hydrolysis of DFP was gradually increased with the increasing TEA concentrations, kinetically suggesting the reduction of fluoride inhibition by the adding of TEA.

**Fig 10:**
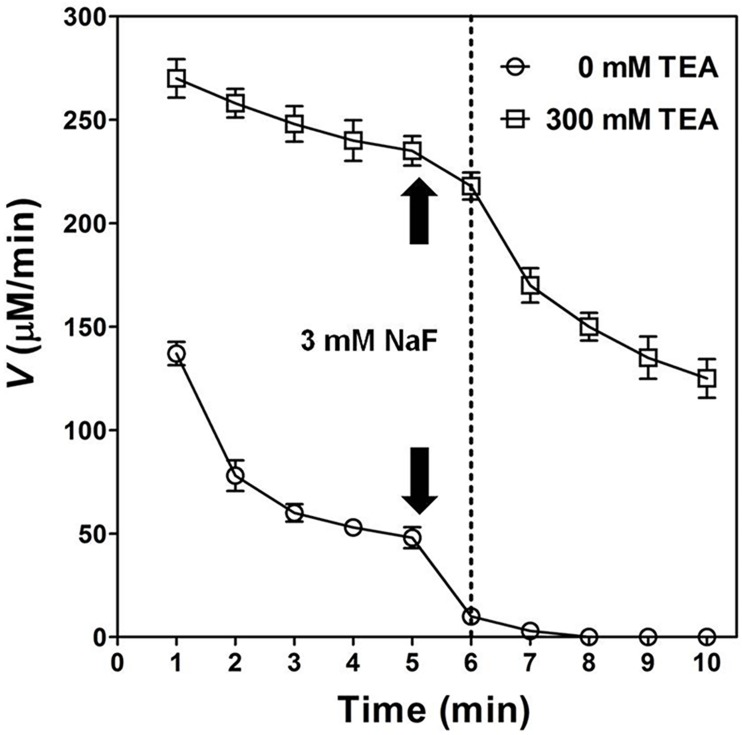
Effect of triethanolamine on inhibition of OPH-catalyzed hydrolysis of DFP by fluoride (NaF) given at a later reaction stage. The velocity (*V*) for OPH-catalyzed hydrolysis of DFP under 0 (○) and 300 mM (□) TEA condition was determined once per minute with adding of 3 mM NaF (inhibitor) at the end of the first 5 minutes, i.e. at the beginning of 6^th^ minute in total reaction time (10 minutes). The DFP concentration used was 20 mM, and the other reaction conditions and statistics analysis were the same as described in Fig 4. The black arrows (up and down) both indicated the time point to add NaF with final concentration (3 mM), and the dotted vertical line in the diagram indicated the first time-point to make record after NaF adding.

**Fig 11:**
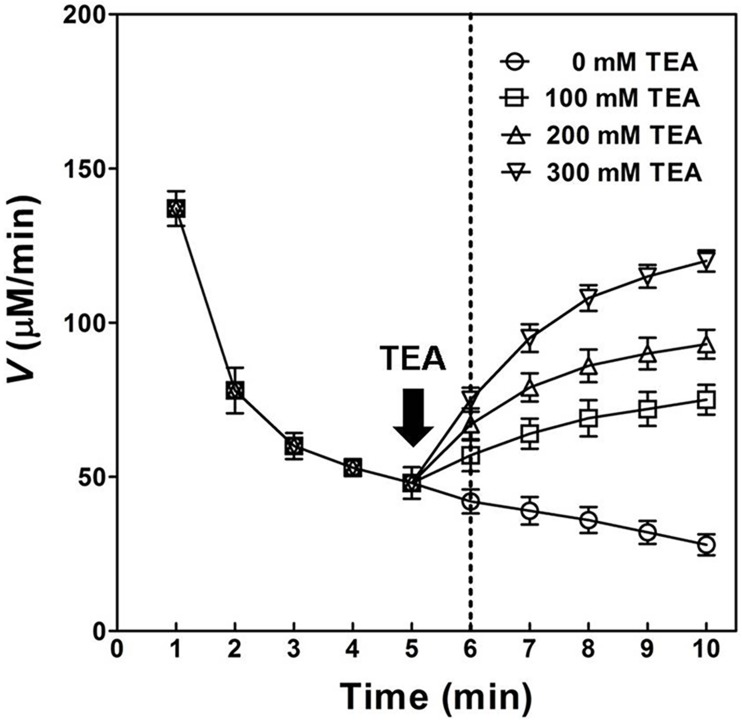
Effect of adding aminoalcohole at a later stage (6–10 minutes) of OPH-catalyzed hydrolysis of DFP. The velocity (*V*) for OPH-catalyzed hydrolysis of DFP was determined once per minute with adding 0 (○), 100 (□), 200 (△), and 300 mM (▽) TEA at the end of the first 5 minutes, i.e. at the beginning of 6^th^ minute in total 10 minutes reaction time. The DFP concentration used was 20 mM, and the other reaction conditions and statistics analysis were the same as described in Fig 4. The black arrow (down) indicated the time point to add TEA with defined final concentrations, and the dotted vertical line in the diagram indicated the first time-point to make record after TEA adding.

In the absence of TEA, the velocity (*V*) for OPH-catalyzed hydrolysis of DFP was decreased more quickly than that in the presence of 300 mM TEA, especially at a later reaction stage (6–10 minutes) with adding of 3 mM NaF inhibitor (Fig 10). As shown in Fig 10, the adding of 3 mM NaF to TEA-free solution at the end of the first 5 minutes reaction time led to total inhibition of OPH towards DFP in the following 2 minutes. In contrast, the *V* for DFP hydrolysis in OPH solution decreased much slower at 300 mM TEA during the whole reaction time (10 minutes). At a later reaction stage (6–10 minutes), the adding of 3 mM NaF to TEA-containing solution exhibited a much less inhibition against OPH activity than that to TEA-free solution (Fig 10), suggesting an obvious "buffering capacity" of the aminoalcohol for fluoride. On the other hand, an obvious rate-enhancing effect on OPH catalysis towards DFP was observed with the adding of 300 mM TEA at the end of the first 5 minutes when the DFP hydrolysis was largely inhibited (Fig 11).

## Discussion

Organophosphorus hydrolase (OPH; EC 3.1.8.1) can hydrolyze a variety of organophosphorus (OP) compounds with P–O, P–S, P–F, and P–CN bonds [1–3,8,23,31,33]. However, this enzyme exhibited much lower activities towards certain OP substrates containing P-F bond, the reactive site in fatal nerve agents sarin (GB), soman (GD) and diisopropylfluorophosphate (DFP) [1–3,6,8,23]. Directed evolution and rational protein engineering have created desirable OPH mutants with enhanced activities to cleave the P-F bond [2,3,16]. However, choice of buffer to further enhance OPH activity towards organophosphofluoridates was rarely reported. Previous studies have documented the activation of bovine intestine alkaline phosphatase (EC 3.1.3.1) to degrade *p*-nitrophenyl phosphate (pNPP) in the presence of aminoalcohol and amine buffers [18–20]. To our knowledge there have been no reports on the effect of reaction buffer on OPH activity towards DFP, a G-type nerve agents analog. The present study reported the aminoalcohol-induced activation of OPH, as illustrated by increase of kinetic parameters *k*_cat_, *V*_max_ and *k*_cat_/*K*_m_ on DFP hydrolysis (Table 1).

The *k*_cat_ values of OPH in the hydrolysis of DFP elevated with increasing concentrations of MEA, DEA, and TEA (Table 1), indicating that they could activate OPH and might be suitable buffers to effectively detoxify the fluoride-containing nerve agent. The magnitudes of the activation by different aminoalcohols, indicated by the *V* and *k*_cat_, were ordered in TEA, DEA, and MEA (Fig 4), revealing the association of the observed activating effects with hydroxyl or hydroxyethyl group concentrations. However, alcohols, including ethanol, glycerol, and methanol, could not activate OPH (Table 2), suggesting that the hydroxyl or hydroxyethyl group alone contributes little to the observed OPH activation. In contrast, all the amines investigated, including methylamine, ethylamine, diethylamine, and triethylamine, had slightly activating effects on OPH activity, as observed in the change of *k*_cat_ and *V*_max_ at 300 mM concentration (Table 3), indicating the critical role of alkyl-substituted amine (amino nitrogen) in the OPH activation. In recent years, a series of chemically synthetic OP scavengers based on organic nitrogen derivatives, including Zn^2+^-azamacrocyclic complexes [21,34], aminopyridine-substituted polyallylamine [35] and oxime-substituted cyclodextrins [22,36–38], have been established to eliminate pesticides and nerve agents such as parathion, paraoxon, DFP, cyclosarin (GF) and tabun. The mechanism of the reported non-enzyme catalysts exclusively involves essential nitrogen atom(s) with lone pair electrons that function as critical Lewis base to initiate catalysis. In the present study, the OPH activation mediated by amines and aminoalcohols was proposed to share the similar mechanism that the amino nitrogen in these activators could participate in the catalytic hydrolysis. On the other hand, at 300 mM, the magnitudes of the activation in *k*_cat_ by diethylamine (1.4 fold) and triethylamine (1.3 fold) are not remarkable compared to that by DEA (2.4 fold) and TEA (3.6 fold), respectively (compare Tables 1 and 3), suggesting that the activating effects of aminoalcohols could be ascribed to the combined effects of the ethyl-substituted amino nitrogen and hydroxyl group. Further experiments indicated the requirement for coexistence of amine and alcohol groups in the same molecule regarding the observed rate-enhancing effects (Table 4). Moreover, such activating effects only occurred for the aminoalcohol additives with two carbon (C) atoms between nitrogen (N) and oxygen (O), but not for 3-amino-1-propanol with more than two C atoms between N and O (Table 5), suggesting some anchimeric assistance in the rate-enhancing. A similar system containing thiodiglycol rather than thiodipropanol was also rate-enhancing (Table 5), confirming the putative anchimeric assistance. Here, a proper carbon-atom distance between two functional groups in aminoalcohol and its stimulant molecule was emphasized for this additive-induced activation of OPH towards DFP.

The proposed catalytic mechanism for OPH based on X-ray structure resolution suggests a critical role of an activated water molecule in the initial nucleophilic attack on the phosphoryl center of the OP substrates [2–4,8–11]. In the reported biochemical process, the activated water molecules have been identified to be hydroxide ions that are stabilized by metal ions (a pair of Zn^2+^) in the enzyme active site [2,4,8,10]. According to the mechanism, the hydroxyl groups in aminoalcohols are likely to generate additional hydrogen bonds that would further stabilize the activated water molecules and/or phosphorus-centered transition state and thus make a faster DFP hydrolysis. Previous studies have described that the introduction of potential hydrogen bond donors to the leaving group pocket of OPH, i.e. the single site mutants F132Y and L140Y, led to substantial improvements in the rates of phosphofluoridate hydrolysis [2,3]. In the present study, the additives with hydrogen bond donors, including amino-N and hydroxyl-O in aminoalcohols (Fig 3), also enhanced the OPH activities to considerably higher levels (Table 1), also reflecting the contribution of hydrogen bond donors to the OPH activation. Another explanation for the aminoalcohol-induced activation could be a favorable hydrophilic environment created by the chemical compounds that allow for efficient fluoride (F^−^) releasing. As previously reported, based on theoretical simulation and experimental mutagenesis, a proper environment transition in active site, hydrophobic to hydrophilic, desirably activated DFPase to cleavage P-F bond with a more efficient fluoride releasing [39,40], achieving a quadruple mutant of DFPase with significantly higher DFP-degrading activity (*k*_cat_/*K*_m_ 1.4 × 10^5^ M^−1^ s^−1^) [40]. In the current aminoalcohol-induced activation of OPH, with the increase of *k*_cat_ and *k*_cat_/*K*_m_, the apparent *K*_m_ values were also increased (Table 1), indicating little contribution of aminoalcohol to facilitating substrate binding. This might mean the reported activating effects of aminoalcohols occurred at the product (leaving group F^−^) releasing step, rather than the DFP (substrate) binding step. Actually, the activation of DFPase towards DFP hydrolysis was predominantly attributed to an easier fluoride leaving [40], rather than an enhanced substrate binding [41]. As an additional evidence, the activating effects of aminoalcohols on OPH catalysis were also observed for diethyl fluorophosphate, the substrate with fluoride as leaving group, but not for ethyl-paraoxon, the substrate with phenolic compound as leaving group (data not shown). Thus, the acceleration of F^−^ releasing in the aminoalcohol buffers might require proper interaction(s) between the hydrogen-bond donors and the hydrophilic leaving groups. The similar activating effects of aminoalcohols have been reported in the BIALP-catalyzed hydrolysis of pNPP with phosphate (Pi) as leaving group [18], also suggesting the specific interaction of aminoalcohols with the hydrophilic leaving groups.

The binuclear metal center in OPH has been evaluated to be essential for the activated hydroxide formation and catalysis initiation [2,4,14]. Dumas et al. [42] have reported the inactivation of OPH by nitrogen-containing compounds of metal chelators including *o*-phenanthroline, EDTA, and 2,6-pyridine dicarboxylate. In the present study, EDTA has been proved to predominantly abolish the TEA-induced activation of OPH (wild-type and mutants) regarding DFP hydrolysis (Fig 6), suggesting the important role of metal center in the observed OPH activation. On the other hand, the previously reported OPH mutants F132Y and L140Y, with the best DFP-degrading activities in the past [2,3], have been validated to be further activated in the TEA-containing buffers (Table 6). The mutated amino acid residues have been emphasized to be potential hydrogen bond donors in or around DFP-binding site for the optimum OPH activities [2,3]. Thus, the TEA-induced activation of OPH observed in this study could not be fully explained by the contribution of hydrogen-bond donors distributed in the aminoalcohol molecules. Here, we deduced that the activating effect of TEA might be associated with certain Zn^2+^ metal center, the specific active center component other than DFP-binding site. Fluoride (F^−^) has been documented as an effective OPH inhibitor by interfering with metal center required in catalysis initiation [14]. The similar fluoride inhibition was observed in the present study (Fig 7 and Table 7), which was further classified as a linear mixed inhibition (Fig 9A). In the OPH-catalyzed hydrolysis of DFP, the product inhibition could be in part released with TEA adding (Fig 8). The *K*_i_ analysis of fluoride did illustrate a gradual decrease in the product inhibition against OPH-catalyzed hydrolysis of DFP with the increasing TEA concentrations (Fig 9). Based on the kinetics data, we suggested that the aminoalcohol-induced activation of OPH may be associated with the reduced fluoride inhibition. In addition, the increase of *K*_i_ for F^−^ (Fig 9) as well as *K*_m_ for the substrate (Table 7) may indicate a slow formation of some certain F^−^-metal complex in the OPH active-site pocket. The current kinetics data, collected in rapid equilibrium conditions (2 h of enzyme-inhibitor incubation), did give a mixed inhibitor behavior, suggesting a possible antagonistic effects of TEA and fluoride in the enzyme metal center. This class of antagonistic effects were also observed in the "buffering capacity" of 300 mM TEA against fluoride inhibitor given at a later OPH reaction stage (Fig 10) and in the predominant velocity recovery for DFP hydrolysis by the adding of 300 mM TEA at the same reaction phase (Fig 11), both partially indicating the involvement of metal center in the TEA-induced relief of fluoride inhibition in the OPH catalysis. Nevertheless, more detail knowledge on the metal-TEA interaction is still not clear in the current study, and some further analysis regarding the enzyme stability with fluoride might be required to uncover the mechanism involved in the TEA-induced rate-enhancing.

The application of OPH in organophosphofluoridate intoxication requires desirable hydrolase activity to cleavage P-F bond. However, the catalytic efficiencies *k*_cat_/*K*_m_ of OPH towards fluoride-containing nerve agents including DFP are ranging from 1.0 × 10^4^ to 8.0 × 10^4^ M^-1^ s^-1^, much lower than that towards its optimal substrate ethyl-paraoxon (*k*_cat_/*K*_m_ = 5.5 × 10^7^ M^-1^ s^-1^) [8,11,39,43]. To date, the desirable enzyme(s) to degrade DFP has been recorded as squid diisopropylfluorophosphatase (DFPase; EC 3.1.8.2) with *k*_cat_/*K*_m_ 1.4 × 10^5^ M^−1^ s^−1^ [11,13,39,40,41] and *Alteromonas* organophosphorus acid anhydrolases (OPAA; EC 3.1.8.2) with about one order of magnitude higher *k*_cat_/*K*_m_ (5.5 × 10^5^ M^−1^ s^−1^) [44–47]. In the present study, the optimal OPH activity towards DFP has been assessed by *k*_cat_/*K*_m_ 541.7 ± 12.6 mM^-1^ s^-1^ (approximately 5.4 × 10^5^ M^-1^ s^-1^) at 300 mM TEA condition (Table 6), desirably higher than DFPase and reaching at the level of OPAA, the best DFP-degrading enzyme ever reported. OPAA has been well exploited to detoxify fluoride-containing OPs and successfully encapsulated as dentritic-enzyme complexes with pralidoxime and/or atropine to protect AChE and/or cholinesterase against DFP [47–49]. In contrast, despite of having a wide OPs spectrum, OPH has been not so good to degrade organophosphofluoridates including DFP, somewhat reducing the practical value of this enzyme. It would be interesting to investigate if the aminoalcohols currently studied also have rate-enhancing effects on the more DFP-specific enzymes including OPAA and DFPase. Nevertheless, based on the current report, the activation of OPH by aminoalcohol buffers would be worthy of consideration to increase efficiency of OPH in the detoxification of organophosphofluoridates.

### Conclusion

Monoethanolamine, diethanolamine, triethanolamine and other aminoalcohols with two carbon atoms between amino nitrogen and hydroxyl oxygen in molecular structure are able to activate OPH towards DFP hydrolysis, and the triethanolamine with the best activating effect might be suitable as a reaction buffer of OPH to rapidly detoxify the nerve agent with P-F bond. Our results also suggest the combined contribution of amino nitrogen and ethoxy group in the reported OPH activation. Further kinetic inhibition studies correlate the activating effect of aminoalcohol with the reduced fluoride inhibition in the DFP hydrolysis. The present work has put forward a buffer choice to enhance OPH activity to degrade fluoride-containing nerve agent, which might be beneficial to design organophosphofluoridate bioscavengers based on OPH catalysts.

## Supporting Information

S1 Fig Identification of chemically synthesized DFP by Nuclear Magnetic Resonance (NMR) spectroscopy. A: ^1^H NMR spectroscopy; B: ^13^C NMR spectroscopy; C: ^19^F NMR spectroscopy; D: ^31^P NMR spectroscopy.
(TIF)

S1 Table Primers used in *opd* gene sub-cloning and mutagenesis. D_29_-OPH represented the leader-sequence-deleted OPH in which 29 N-terminal amino acids had been removed. The truncated version of OPH was designated as the wild-type enzyme in this study. 1 and 2 indicated the forward and reverse primer, respectively. PCR products using the primer pairs were sub-cloned into pET-28 to construct expression plasmids.
(PDF)

## Abbreviations

AChE: acetylcholinesterase
ALP: alkaline phosphatase
BIALP: bovine intestine ALP
DEA: diethanolamine
DFPase: diisopropylfluorophosphatase
DFP: diisopropylfluorophosphate
EDTA: ethylenediaminetetraacetate
GB: sarin
GD: soman
GF: cyclosarin
IPTG: Isopropyl thio-β-dgalactoside
MEA: monoethanolamine
NMR: Nuclear Magnetic Resonance
OPH: organophosphorus hydrolase
OP: organophosphorus
OPs: organophosphorus pesticides
OPAA: organophosphorus acid anhydrolase
PTE: phosphotriesterase
pNPP: *p*-nitrophenyl phosphate
PAPs: purple acid phosphatases
SDS-PAGE: sodium dodecyl sulfate polyacrylamide gel electrophoresis
TEA: triethanolamine
