# Supplementary figures and images for: Aminoalcohol-Induced Activation of Organophosphorus Hydrolase (OPH) towards Diisopropylfluorophosphate (DFP)
Source: PLoS One. 2017 Jan 13;12(1):e0169937. doi: 10.1371/journal.pone.0169937 (PMC5234802; doi:10.1371/journal.pone.0169937)

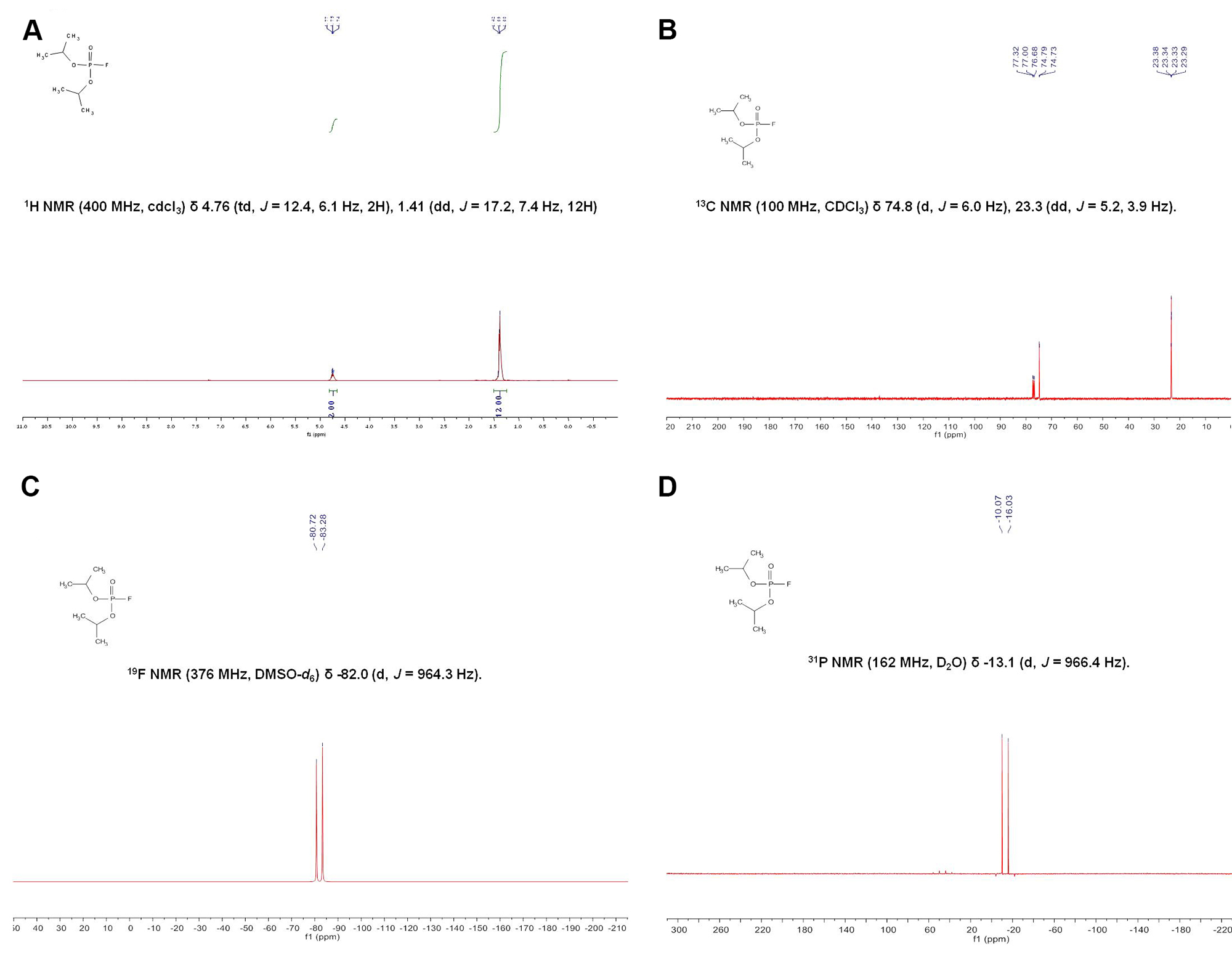

Supplement: S1 Fig — A: 1H NMR spectroscopy; B: 13C NMR spectroscopy; C: 19F NMR spectroscopy; D: 31P NMR spectroscopy. (TIF) [file pone.0169937.s001.tif]
