# Supplementary material for: Aminoalcohol-Induced Activation of Organophosphorus Hydrolase (OPH) towards Diisopropylfluorophosphate (DFP)
Source: PLoS One. 2017 Jan 13;12(1):e0169937. doi: 10.1371/journal.pone.0169937 (PMC5234802; doi:10.1371/journal.pone.0169937)
Supplement: S1 Table — D29-OPH represented the leader-sequence-deleted OPH in which 29 N-terminal amino acids had been removed. The truncated version of OPH was designated as the wild-type enzyme in this study. 1 and 2 indicated the forward and reverse primer, respectively. PCR products using the primer pairs were sub-cloned into pET-28 to construct expression plasmids. (PDF) [file pone.0169937.s002.pdf]

S1 Table. Primers used in *opd* gene sub-cloning and mutagenesis. D_{29}-OPH represented the leader-sequence-deleted OPH in which 29 N-terminal amino acids had been removed. The truncated version of OPH was designated as the wild-type enzyme in this study. 1 and 2 indicated the forward and reverse primer, respectively. PCR products using the primer pairs were sub-cloned into pET-28 to construct expression plasmids.

Primer	Sequence (5’→3’)	Restriction enzyme
D_{29}-OPH-1	GGAATTCATGTCGATCGGCACAGGCG	*Eco*RI
D_{29}-OPH-2	CCCAAGCTTTCATGACGCCCGCAAGG	*Hin*dIII
F132Y-1	CTTGTGGTACGACCCGCCACTTTC	None
F132Y-2	TAAGTGGCGGGTCGTACCACAAGC	None
L140Y-1	TACGATGCGATATAGGAGTGTAGAGG	None
L140Y-2	GTTCCTCTACACTCCTATATCGCATC	None
